# Telomere-to-telomere genome assembly of matsutake (*Tricholoma matsutake*)

**DOI:** 10.1093/dnares/dsad006

**Published:** 2023-04-25

**Authors:** Hiroyuki Kurokochi, Naoyuki Tajima, Mitsuhiko P Sato, Kazutoshi Yoshitake, Shuichi Asakawa, Sachiko Isobe, Kenta Shirasawa

**Affiliations:** Department of Forest Science, Graduate School of Agricultural and Life Sciences, The University of Tokyo, Tokyo 113-8657, Japan; Department of Frontier Research and Development, Kazusa DNA Research Institute, Kisarazu, Chiba 292-0818, Japan; Department of Frontier Research and Development, Kazusa DNA Research Institute, Kisarazu, Chiba 292-0818, Japan; Department of Aquatic Bioscience, Graduate School of Agricultural and Life Sciences, The University of Tokyo, Tokyo 113-8657, Japan; Department of Aquatic Bioscience, Graduate School of Agricultural and Life Sciences, The University of Tokyo, Tokyo 113-8657, Japan; Department of Frontier Research and Development, Kazusa DNA Research Institute, Kisarazu, Chiba 292-0818, Japan; Department of Frontier Research and Development, Kazusa DNA Research Institute, Kisarazu, Chiba 292-0818, Japan

**Keywords:** genome assembly, long, read sequencing technology, telomere, to, telomere

## Abstract

Here, we report the first telomere-to-telomere genome assembly of matsutake (*Tricholoma matsutake*), which consists of 13 sequences (spanning 161.0 Mb) and a 76 kb circular mitochondrial genome. All the 13 sequences were supported with telomeric repeats at the ends. GC-rich regions are located at the middle of the sequences and are enriched with long interspersed nuclear elements (LINEs). Repetitive sequences including long-terminal repeats (LTRs) and LINEs occupy 71.6% of the genome. A total of 21,887 potential protein-coding genes were predicted. The genomic data reported in this study served not only matsutake gene sequences but also genome structures and intergenic sequences. The information gained would be a great reference for exploring the genetics, genomics, and evolutionary study of matsutake in the future, and ultimately facilitate the conservation of this vulnerable genetic resource.

## 1. Introduction

Matsutake (*Tricholoma matsutake* [S. Ito et Imai] Singer), belonging to the phylum Basidiomycota, is an ectomycorrhizal fungus that coexists with Pinaceae and Fagaceae trees in a symbiotic association.^1,2^ In the field, two spores of matsutake fuse together and grow to form a ‘shiro’, which is a symbiotic entity formed between matsutake and its host tree. One shiro produces a number of sporocarps during the growing season. The sporocarp of matsutake has been considered as one of the most valuable components of traditional Japanese cuisine since ancient times, as mentioned in Manyo-shu (a series of books for Japanese poetry compiled around 700 AD in Japan), owing to its pleasant aroma, which is largely attributed to 1-octen-3-ol (also known as matsutakeol)^3,4^; however, sporocarps are non-culturable. In 2019, the International Union for Conservation of Nature categorized matsutake as vulnerable. The production of sporocarps has drastically decreased in recent years^5^ because of the deterioration of its growing environment. To understand the life cycle and life history of matsutake, safeguarding its production and conservation is necessary, which requires genomic analysis.

Four assemblies of the matsutake genome are currently available in a public DNA database.^6,7^ However, the sequences are highly fragmented because contigs are enormous in number (2,545–88,884) and short (N50 length = 2.9–320.9 kb), thus providing insufficient genome coverage even though comprehensive protein-coding genes might be represented by the sequences.^6^ Moreover, because retrotransposons such as *MarY1* span ~6 kb in length and are dispersed throughout the matsutake genome,^8^ a full-length genome assembly may not be achieved with short-read and error-prone long-read sequencing technologies, both of which were employed to construct the four genome assemblies. The recent advanced sequencing technologies, e.g. high-fidelity long-read (HiFi) technology (PacBio, Menlo Park, CA, USA) together with ultralong-read sequencing (Oxford Nanopore Technologies, Oxford, UK), PCR-free sequencing (Illumina, San Diego, CA, USA), high-throughput chromosome conformation (Arima Genomics, Carlsbad, CA, USA), optical maps (Bionano Genomics, San Diego, CA, USA), and single-cell DNA template strand sequencing (10X Genomics, Pleasanton, CA, USA), enabled to span repetitive sequences in genomes. These technologies contribute to establish complete gapless assemblies of the human haploid genome at the telomere-to-telomere level,^9^ in which a single contig corresponds to a single chromosome.

In this study, we applied the HiFi technology to address the complexity of the matsutake genome. Using this technology, we established 13 telomere-to-telomere sequences, which would provide not only matsutake gene sequences but also the genome structures and the intergenic sequences. Overall, this study represents a milestone in the cytogenetics-, genetics-, and genomics-focussed research on matsutake mushroom.

## 2. Materials and methods

### 2.1. Fungus material and DNA extraction

Two sporocarps, which were probably ramets derived from a single shiro (radius > 2 m) that has been generating sporocarps for more than 20 years,^10^ were collected from Ina, Nagano, Japan. The sporocarps were flash-frozen in liquid nitrogen, dried under vacuum, and then stored at room temperature until needed for DNA extraction.

Genomic DNA was extracted from the dried stipes using the cetyltrimethylammonium bromide (CTAB) method.^11^ The concentration of the extracted DNA was measured using the Qubit dsDNA BR assay kit (Thermo Fisher Scientific, Waltham, MA, USA), and DNA fragment length was evaluated by agarose gel electrophoresis with Pippin Pulse (Sage Science, Beverly, MA, USA).

### 2.2. DNA sequencing

Genomic DNA was subjected to HiFi SMRTbell library construction using the SMRTbell Express Template Prep Kit 2.0 (PacBio), according to the manufacturer’s instructions, with a minor modification. Because the genomic DNA was degraded, the DNA shearing step recommended in the protocol was skipped. The resultant DNA was fractionated with BluePippin (Sage Science) to eliminate fragments less than 10 kb in size. The DNA libraries prepared from the two sporocarps were indexed with unique barcode adapters, and sequenced on a single SMRT cell 8M on the Sequel IIe system (PacBio).

### 2.3. Genome assembly and gene annotation

Using the HiFi reads obtained from the Sequel IIe system (PacBio), the genome size and heterozygosity of matsutake was estimated with GCE^12^ and GenomeScope,^13^ respectively, based on *k*-mer frequency (*k* = 21) calculated with Jellyfish^14^ (version 2.3.0). The reads were assembled using hifiasm^15^ (version 0.16.1), with default parameters or with the primary mode. Assembly completeness was evaluated with Benchmarking Universal Single-Copy Orthologs (BUSCO)^16^ (version 5.2.2; default parameters) using lineage dataset agaricales_odb10 (eukaryota, 2020-08-05). Telomere sequences containing repeats of a 6 bp motif (5ʹ-TTAGGG-3ʹ) were searched by the search subcommand of tidk (https://github.com/tolkit/telomeric-identifier) with a window size of 10,000. Contig connections were evaluated with D-GENIES^17^ or PCR as described by Shirasawa *et al*.^18^ with a pair of primers (5ʹ-TTTGCTGGAAACACAGTAAACTACA-3ʹ and 5ʹ-CTGGGTAATCTTGTGAAACTCTGTC-3ʹ). Amplified DNA was electrophoresed with Genomic DNA ScreenTape on 4200 TapeStation System (Agilent Technologies, Santa Clara, CA, USA). Potential contaminated sequences were identified by a sequence similarity search to UniProtKB^19^ using DIAMOND^20^ with an *E*-value cutoff of <1E-10.

Nuclear genes were predicted with GeMoMa (version 1.9)^21^ by mapping predicted genes in the matsutake genome, Trima3,^6^ with MMseqs.^22^ Mitochondrial genes were predicted with Artemis,^23^ in accordance with the gene sequences reported in previous mitochondrial genome assemblies (accession number: NC_028135). The predicted genes were functionally annotated with emapper^24^ (version 2.1.6; search option: mmseqs) implemented in EggNOG,^25^ and with DIAMOND^20^ (version 2.0.13; more sensitive mode) search against the UniProtKB^19^ database. Repetitive sequences in the assembly were identified with RepeatMasker (https://www.repeatmasker.org) (version 4.1.2; parameters: -poly and -xsmall) using repeat sequences registered in Repbase^26^ and a *de novo* repeat library built with RepeatModeler (https://www.repeatmasker.org) (version 2.0.2a; default parameters). Sequences showing similarity to *MarY1* (accession number: AB028236; 6047 bp) and its long terminal repeats (LTRs; 426 bp) were searched by BLASTN.^27^

## 3. Results

### 3.1. DNA sequencing, data analysis, and genome assembly

Genomic DNA was extracted from two dried sporocarps (samples A and B) of matsutake. The amount of DNA extracted from each sample (9 µg) was sufficient for library construction; however, because of degradation (Supplementary Fig. S1), the extracted DNA was used for library preparation without shearing. The resultant libraries were sequenced on a SMRT Cell 8M to obtain 9.5 Gb (sample A) and 7.8 Gb (sample B) data, with N50 lengths of 11 kb (sample A) and 10 kb (sample B). The *k*-mer analysis detected two peaks (Supplementary Fig. S2), indicating that the haploid genome size of matsutake was 149 Mb and the level of heterozygosity was 1.05%. The sequence reads of each sample were assembled separately to obtain two sets of contigs: 182 contigs (165.5 Mb) for sample A, and 146 contigs (162.9 Mb) for sample B (Supplementary Table S1). In parallel, haplotype phased contigs were generated for samples A and B (Supplementary Table S1): 163.3 Mb in the haplotype 1 of sample A (Ahap1); 158.8 Mb in Ahap2; 163.8 Mb in Bhap1; and 157.0 Mb in Bhap2.

**Figure 1.**
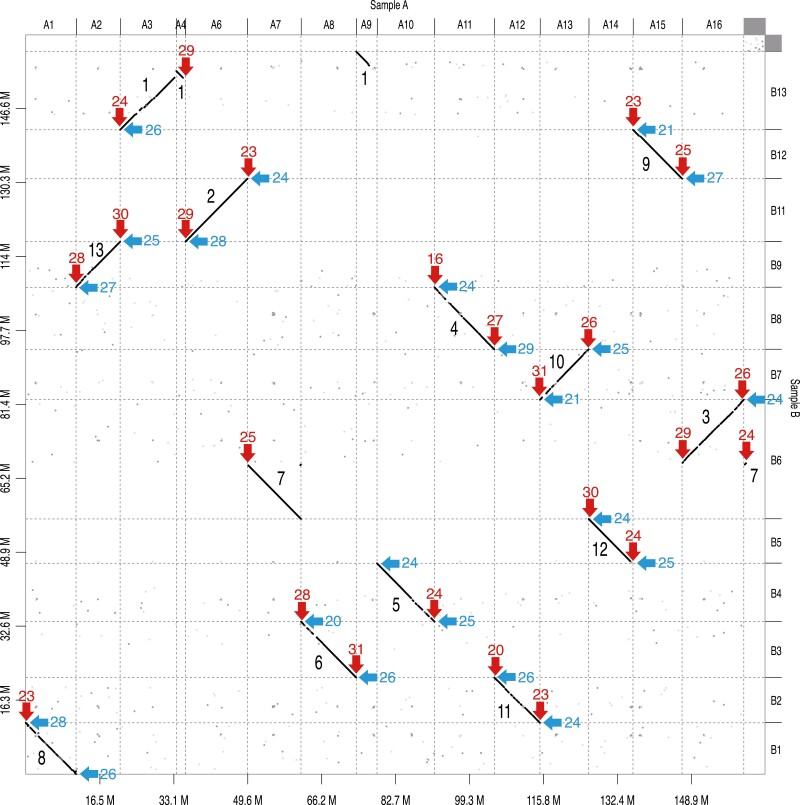
Comparative map of contigs of samples A and B. Dots indicate sequences with a sequence identity of ≥75% between the two samples. Red and blue arrows indicate telomeric motifs detected at the ends of contigs of samples A and B, respectively. Black numbers in the plot indicate sequence names in the final assembly (TMA_r1.1) and red and blue numbers by the arrows indicate repeat numbers of telomeric motifs. Contigs A5 and B10 are lacked because of the short sequence length (<1 Mb).

Next, we searched for the telomeric motif, (TTAGGG)n, in the contigs (Fig. 1). In sample A, the telomeric motif was found at both ends of nine contigs (A2, A6, A8, A11, A12, A13, A14, A15, and A16) and at one end of six contigs (A1, A3, A4, A7, A10, and A22). In sample B, the motif was found at both ends of 10 contigs (B1, B2, B3, B4, B5, B7, B8, B9, B11, and B12) and at one end of two contigs (B6 and B13). The length of the telomeric sequence was ranged from 96 bp (16 repeats) to 186 bp (31 repeats).

Comparison of the two sets of genome assemblies revealed 10 pairs of aligned contigs (A1-B1, A2-B9, A6-B11, A8-B3, A10-B4, A11-B8, A12-B2, A13-B7, A14-B5, and A15-B12) (Fig. 1). Three contigs of sample A (A3, A4, and A9) covered the entire sequence of one contig of sample B (B13). Furthermore, three contigs of sample A (A7, A16, and A22) corresponded to one contig of sample B (B6). Thus, we assumed that contigs A3, A4, and A9 were unassembled, and contig B6 was misassembled at the telomeres. Therefore, we joined contigs A3, A4, and A9 with 100 Ns to establish a single contig, and left contigs A7 and A22 were separated from A16. The jointed points between A7 and A22 and between A4 and A9 were supported by haplotype phased contigs (Supplementary Fig. S3A), and the remaining points between A3 and A9 was verified with PCR (Supplementary Fig. S3B).

**Figure 2.**
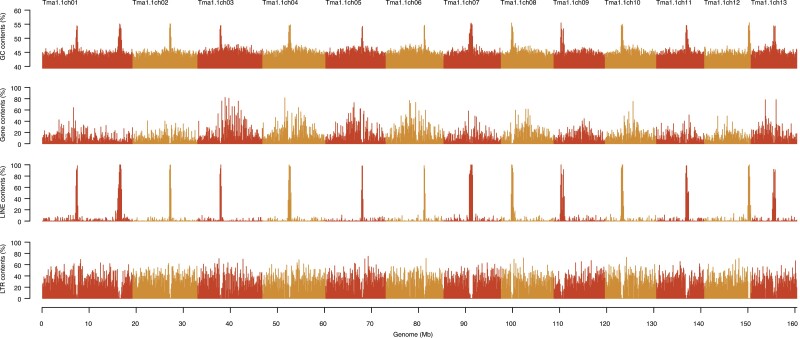
Features of the matsutake genome. Bars indicates the GC content and numbers of genes, LINEs, and LTRs within a 100 kb window.

Finally, 13 contigs spanning 161.0 Mb were obtained, all which contigs were supported with telomeric motifs at both ends. The remaining contigs as potential contaminated sequences were eliminated from the following analysis because of no sequence similarity with nuclear protein-coding genes of Trima3. The 13 contigs represented 94.2% complete BUSCOs. The final assembly was designated as TMA_r1.1, and the contigs were named TMA_r1.1ch01 to TMA_r1.1ch13 in order of decreasing sequence length (Fig. 1, Table 1). The GC content was ca. 45% over the entire genome, with one peak (~55%) in each chromosome, except chromosome 1, which showed two peaks (Fig. 2). In addition, we identified a 76,067 bp circular contig, which represented the mitochondrial genome of matsutake (Tma1.0mito).

**Table 1.** Statistics of the matsutake genome assembly

Chromosome	Sequence length (bp)	No. of genes	Contigs of sample A	Contigs of sample B
Tma1.0ch01	19,249,005	2,139	A3, A4, A9	B13
Tma1.0ch02	13,874,649	1,625	A6	B11
Tma1.0ch03	13,809,479	2,285	A16	B6 (bottom)
Tma1.0ch04	13,409,878	2,032	A11	B8
Tma1.0ch05	12,860,286	1,968	A10	B4
Tma1.0ch06	12,376,747	1,991	A8	B3
Tma1.0ch07	12,297,795	1,421	A7, A22	B6 (top)
Tma1.0ch08	11,264,506	1,594	A1	B1
Tma1.0ch09	10,996,241	1,368	A15	B12
Tma1.0ch10	10,915,969	1,520	A13	B7
Tma1.0ch11	10,203,996	1,247	A12	B2
Tma1.0ch12	9,912,917	1,205	A14	B5
Tma1.0ch13	9,869,253	1,492	A2	B9
**Total**	**161,040,721**	**21,887**		

### 3.2. Repetitive sequence analysis

Repetitive sequences occupied a total physical distance of 115.2 Mb (71.6%) in the genome assembly (TMA_r1.1; 161.0 Mb). Nine major types of repeats were identified in varying proportions (Table 2). The dominant repeat types in the chromosome sequences were LTRs (60.2 Mb) and long interspersed nuclear elements (LINEs; 8.9 Mb). LINEs were predominant in regions with high GC content in all chromosomes, whereas LTR retrotransposons were predominant in regions with low GC content (Fig. 2). Repeat sequences unavailable in public databases totalled 40.9 Mb. Among the LTR retrotransposons, *MarY1*, which has been extensively studied to date, and its terminal repeats were present as 683 and 3,240 copies, respectively, across all 13 chromosomes.

**Table 2.** Repetitive sequences in the matsutake genome

Type of repetitive sequence	Copy number	Length (bp)	Proportion of genome (%)
SINEs	13	757	>0.0
LINEs	7,513	8,876,043	5.5
LTR elements	41,308	60,201,805	37.4
DNA transposons	10,860	7,871,873	4.9
Small RNA	2342	1,936,958	1.2
Satellites	363	74,650	>0.0
Simple repeats	9,342	407,271	0.3
Low complexity	981	48,999	>0.0
Unclassified	111,844	40,948,943	25.4

### 3.3. Gene prediction and annotation

TMA_r1.1 was predicted to contain a total of 21,887 protein-coding genes (Table 1). These predicted genes possessed 93.0% complete BUSCOs. Additionally, sequence alignment revealed that of the 22,885 genes predicted in the previous assembly (Trima3), 22,152 were represented in the current assembly (TMA_r1.1).

## 4. Discussion

This study presents 13 telomere-to-telomere genome sequence of matsutake (Fig. 1, Table 1). In addition to the telomeric repeat motifs at the ends of the sequences, GC-rich regions were found at a single position in all chromosomes, except chromosome 1, which had two GC-rich regions (Fig. 2). Interestingly, the GC-rich regions were enriched with LINEs but devoid of LTRs (Fig. 2). Together, these observations suggest that GC-rich regions might represent centromeres, and that chromosome 1 is likely a dicentric chromosome formed by the telomeric fusion of two chromosomes. We also compared the genome assemblies generated from two independent data sets (samples A and B) (Fig. 1). Consequently, it was possible to identify a misassembled region and an unassembled region (Table 1), which led to the establishment of a telomere-to-telomere genome assembly. To the best of our knowledge, haploid chromosome number of matsutake (*n* = 7) has been reported in only one study to date.^28^ Constructing a telomere-to-telomere assembly could serve as an alternative to karyotyping for proposing the chromosome number of a species, for which no chromosome information is available. Further chromosome observations would be required to validate the assumption and characterize the matsutake chromosomes.

The telomere-to-telomere genome assembly generated in this study spans a physical distance pf 161.0 Mb. Whereas the assembly size was 8% larger than the estimated size of 149.0 Mb (Supplementary Fig. S2), this discrepancy was observed in other organisms depending on species and estimation methods.^29^ The genome size of matsutake is larger than that of other mushroom species^6,7^ because of the high proportion of repetitive sequences (Table 2)^30^. Owing to its high content of repetitive sequences (Table 2) and high heterozygosity (Supplementary Fig. S2), the matsutake genome could not be fully sequenced with short-read and error-prone long-read sequencing technologies. The HiFi sequencing technology (~10 kb read length) employed in this study likely helped overcome the problem posed by repetitive sequences, such as *MarY1* (~6 kb), thus enabling the construction of the telomere-to-telomere genome assembly. Owing to the long contigs and high genome coverage, 21,887 genes were predicted in the matsutake genome.

The genome sequences and predicted genes could help us understand the ecophysiology of a shiro and thus reveal the mechanism of sporocarp formation. The long contiguity sequence would provide not only the matsutake genes but also the genome structure and the intergenic sequences, which could contribute biological and evolutional studies of mushroom. Whole-genome sequencing analysis of matsutake lines would provide sequence differences between alleles and their chromosomal locations. This information could be used to reveal the genetic diversity of matsutake in nature, conserve its genetic resources, and ensure its production. Furthermore, genetic analysis, i.e. genome-wide association study, could reveal the genetic mechanisms underlying phenotypic variations in the physiological and metabolomic traits of matsutake. As mentioned above, the matsutake genome assembly constructed in this study could serve as a reference for further genomics and genetic studies.

## Supplementary Material

dsad006_suppl_Supplementary_Data

dsad006_suppl_Supplementary_Tables

## Data Availability

Raw sequence reads were deposited in the Sequence Read Archive (SRA) database of the DNA Data Bank of Japan (DDBJ) under the accession number DRA014434. Assembled sequences are available at DDBJ (accession numbers AP026538 - AP026551) and Plant GARDEN (https://plantgarden.jp).
