# Supplementary material for: Telomere-to-telomere genome assembly of matsutake (Tricholoma matsutake)
Source: DNA Res. 2023 Apr 25;30(3):dsad006. doi: 10.1093/dnares/dsad006 (PMC10211490; doi:10.1093/dnares/dsad006)
Supplement: dsad006_suppl_Supplementary_Data [file dsad006_suppl_supplementary_data.pdf]

Supplementary Information

Telomere-to-telomere genome assembly of matsutake (*Tricholoma matsutake*)

Hiroyuki Kurokochi[1], Naoyuki Tajima[2], Mitsuhiko P. Sato[2], Kazutoshi Yoshitake[3], Shuichi Asakawa[3], Sachiko Isobe[2], Kenta Shirasawa[2*]

[1]Department of Forest Science, Graduate School of Agricultural and Life Sciences, University of Tokyo, Tokyo 113-8657, Japan

[2]Department of Frontier Research and Development, Kazusa DNA Research Institute, Kisarazu, Chiba 292-0818, Japan

[3]Department of Aquatic Bioscience, Graduate School of Agricultural and Life Sciences, University of Tokyo, Tokyo 113-8657, Japan

[*]To whom correspondence should be addressed:
Kenta Shirasawa
2-6-7 Kazusa-Kamatari, Kisarazu, Chiba 292-0818, Japan
Tel.: +81-438-52-3935
Fax: +81-438-52-3934
E-mail: shirasaw@kazusa.or.jp

Supplementary Table S1 Assembly statistics for the two matsutake samples.

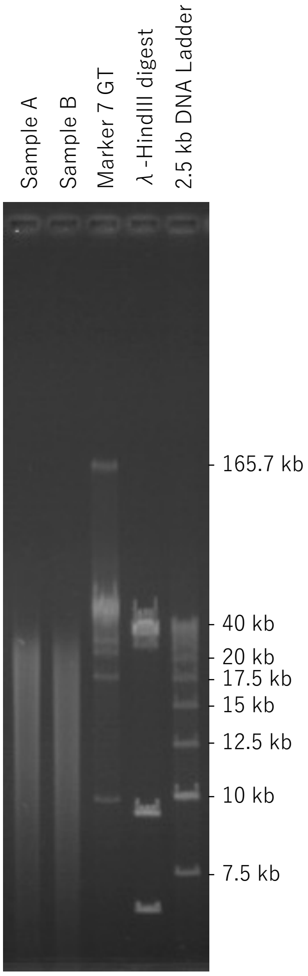

Supplementary Figure S1 Genomic DNA extracted from dried matsutake sporocarps.
Lanes 1 and 2 indicate the genomic DNA of matsutake samples A and B, respectively. The three molecular weight markers used are as follows: Marker 7 GT (Nippongene, Tokyo, Japan), λ-HindIII digest (Thermo Fisher Scientific, Waltham, MA, USA), and 2.5 kb DNA Ladder (Takara Bio, Kusatsu, Japan).

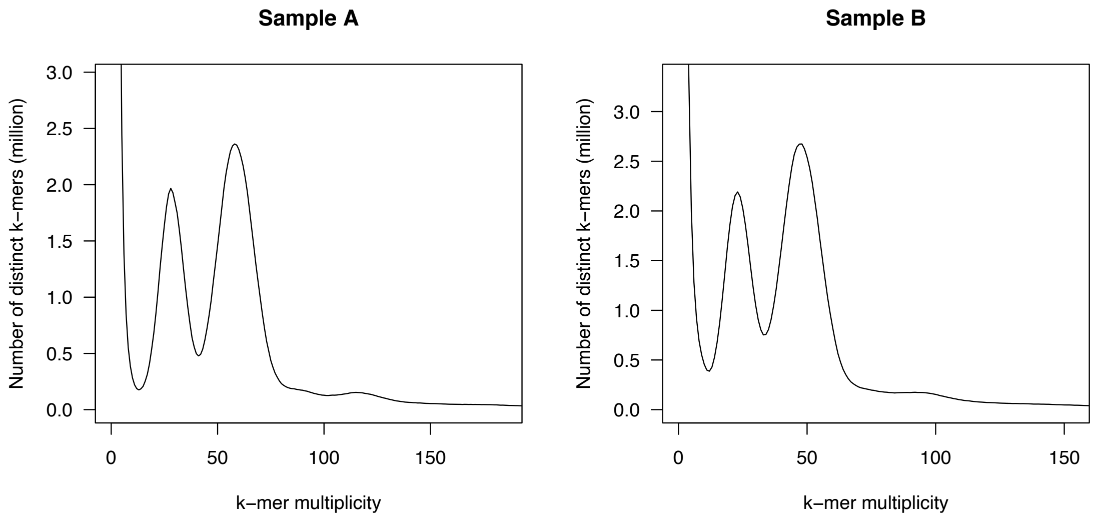

Supplementary Figure S2. Estimation of the genome size of matsutake, based on *k*-mer analysis (k = 21) with the given multiplicity values.

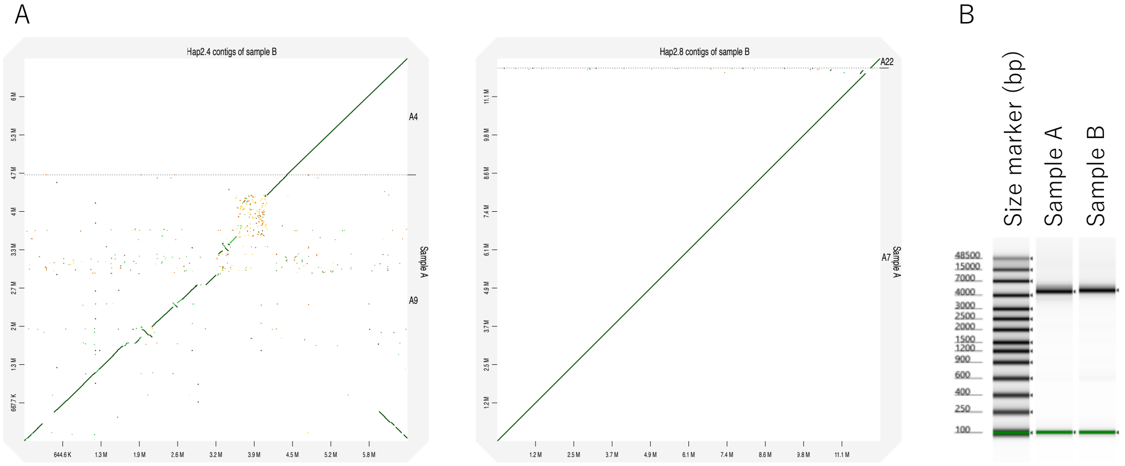

Supplementary Figure S3 Validations of contig connections.

A. Sequence alignment of connected contigs of the sample A and contiguous sequences of the Sample B. Dots indicate sequences with a sequence identity of ≥75% between the two samples. **B.** Amplified DNA from the samples A and B. The primer pairs used in PCR are designed to bridge two contigs.
